# Perioperative Risk of Palliative Gastrectomy in Advanced Gastric Cancer: A Nationwide Multicenter Analysis of Severe Complications and Mortality

**DOI:** 10.3390/cancers18111753

**Published:** 2026-05-27

**Authors:** Sang-Ho Jeong, Miyeong Park, Kyung Won Seo, Inyoung Lee, Jeong Woo Kim, Jae-Seok Min, Sungsoo Park, Information Committee of the Korean Gastric Cancer Association

**Affiliations:** 1 Department of Surgery, Gyeongsang National University College of Medicine and Gyeongsang National University Changwon Hospital, Changwon 51472, Republic of Korea; shjeong@gnu.ac.kr; 2 Department of Anesthesiology, Gyeongsang National University College of Medicine and Gyeongsang National University Changwon Hospital, Changwon 51472, Republic of Korea; 3 Department of Surgery, Kosin University Gospel Hospital, Busan 49267, Republic of Korea; 4 Division of Foregut Surgery, Department of Surgery, Korea University Anam Hospital, Korea University College of Medicine, Seoul 02841, Republic of Korea

**Keywords:** stomach neoplasms, palliative surgery, curative gastrectomy, complications, mortality

## Abstract

Background: Palliative surgery is often considered for advanced stages of gastric cancer to reduce symptoms and improve quality of life; however, it is associated with considerable risks of postoperative complications and mortality. The aim of this study is to analyze the differences in severe complication rates and mortality between palliative and curative gastric cancer surgeries using data from a nationwide survey conducted by the Korean Gastric Cancer Association. Materials and Methods: Data from 12,420 patients who underwent gastric cancer surgery in 2019 were analyzed. Surgical procedures were categorized as total gastrectomies (TGs), distal gastrectomies (DGs), or bypass operations. Patients were divided into curative gastrectomy (CG, *n* = 12,114) and palliative surgery (PS, *n* = 306) groups. Postoperative complications were classified using the Clavien–Dindo (C-D) classification. Severe complications were defined as C-D grade IIIa or higher. Results: Compared with the CG group, the PS group had significantly higher rates of severe complications (10.2% vs. 4.8%, *p* < 0.001) and mortality (1.6% vs. 0.2%, *p* = 0.001). Leakage (3.9% vs. 1.3%, *p* = 0.001) and pancreatic fistula (1% vs. 0.2%, *p* = 0.036) were significantly more common in the PS group. When compared by resection extent, the PS group had higher severe complication rates than the CG group for DGs (13% vs. 3.8%, *p* < 0.001) and a higher mortality rate for TGs (3.3% vs. 0.3%, *p* = 0.006). Conclusions: Palliative gastric cancer surgeries are associated with significantly higher rates of severe complications and mortality than are curative surgeries. These findings emphasize the need for careful patient selection and thorough preoperative counseling when considering palliative gastric cancer surgery.

## Simple Summary

Palliative surgery for advanced gastric cancer may help relieve symptoms such as bleeding or obstruction, but it can also cause serious harm after surgery. This study used a large nationwide dataset from the Korean Gastric Cancer Association to compare short-term outcomes between palliative surgery and curative gastrectomy in patients with gastric cancer. The authors found that patients who underwent palliative surgery had clearly higher rates of severe postoperative complications and death than those who underwent curative surgery. In particular, serious problems such as leakage and pancreatic fistula were more frequent after palliative surgery. The risks were especially high when total gastrectomy was performed. These findings highlight that palliative gastric cancer surgery should be considered very carefully, with strict patient selection and clear preoperative discussion about risks and expected benefits.

## 1. Introduction

Gastric cancer remains one of the leading causes of cancer-related mortality worldwide and has a high prevalence, particularly in East Asia [1,2]. For advanced stages of the disease where curative resection is no longer feasible, palliative surgery is often considered to reduce symptoms, improve quality of life, and manage complications such as bleeding, obstruction, and pain [3,4]. Unlike curative gastric cancer surgeries, which aim to remove all cancerous tissue and achieve long-term survival, palliative surgeries are focused primarily on symptom management rather than eradication of the disease.

While palliative gastric surgery can provide significant relief from tumor-related symptoms, it is also associated with a considerable risk of postoperative complications and mortality, often influenced by the patient’s poor general condition and advanced disease stage [5,6,7]. Complications such as infection, anastomotic leakage, and postoperative bleeding can substantially impact patient outcomes, highlighting the need for careful patient selection and optimized perioperative management. Despite the clinical importance of palliative surgery in the management of advanced gastric cancer, few studies have compared the incidence and nature of complications and mortality rates between palliative and curative gastric cancer surgeries.

The role of palliative gastrectomy in advanced gastric cancer remains controversial [8,9,10,11,12]. The REGATTA trial, a landmark phase III randomized controlled study, challenged the conventional wisdom regarding palliative gastrectomy in advanced gastric cancer patients with a single noncurable factor [9]. This trial revealed no significant survival benefit for gastrectomy followed by chemotherapy compared to chemotherapy alone. However, the REGATTA trial did not directly compare complication rates between palliative and curative gastrectomy operations. The aim of our study is to address this gap in surgical outcomes by analyzing nationwide survey data to compare the complication and mortality rates between palliative and curative gastrectomy procedures.

## 2. Materials and Methods

### 2.1. Study Design and Data Collection

This study analyzed data from a nationwide survey conducted in 2019 by the Korean Gastric Cancer Association (KGCA) [13]. A standardized case report form (CRF) with 54 structured items was distributed to 68 participating institutions. The CRF collected detailed information on patient demographics, comorbidities, tumor characteristics, surgical approaches, and postoperative outcomes. Pathological staging followed the eighth edition of the American Joint Committee on Cancer’s tumor-node-metastasis (TNM) system [14]. Data were validated through quality control checks by the KGCA Information Committee, with discrepancies addressed in consultation with institutional representatives. The data were collected from March to December 2020 [13]. This study was approved by the Institutional Review Board (IRB) at Gyeongsang National University Changwon Hospital (GNUCH 2024-10-022).

### 2.2. Patient Selection and Grouping

**Figure 1:**
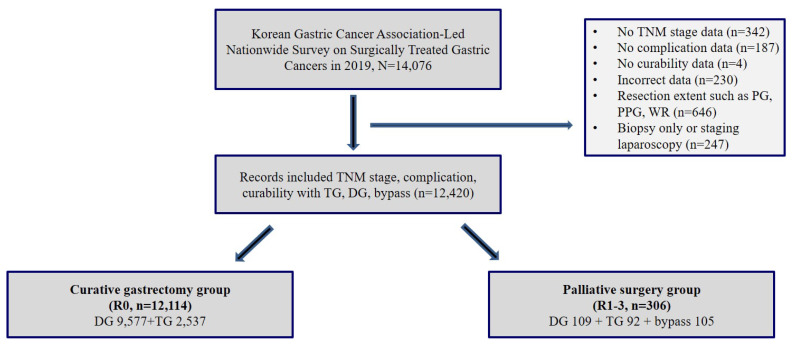
Flow chart of study selection. PG, Proximal gastrectomy; PPG, Pylorus preserving gastrectomy; WR, Wedge resection; DG, Distal gastrectomy; TG, Total gastrectomy.

The study included an initial cohort of 14,076 patients who underwent gastric cancer surgery at 68 participating institutions. Patients with incomplete data regarding tumor staging, postoperative complications, or resection curability were excluded, yielding a final analytic cohort of 12,420 patients (Figure 1). These patients were stratified into two groups based on pathologically determined surgical intent: a curative gastrectomy (CG) group (*n* = 12,114) and a palliative surgery (PS) group (*n* = 306).

Surgical intent was classified according to two objective, pathology-based criteria. Curative intent was defined as complete resection with no residual tumor (R0), corresponding to TNM pathological Stages I–III. Palliative intent was defined as any surgery resulting in microscopic residual disease (R1), macroscopic residual disease at the primary site (R2), or distant metastasis, peritoneal seeding, or positive peritoneal cytology precluding complete resection (R3), irrespective of the extent of gastric resection performed; all such cases corresponded to TNM Stage IV disease. This classification, grounded in pathological staging and resection margin status rather than preoperative clinical intent alone, ensured reproducible and internally consistent group assignment across all participating centers. Surgical procedures were further categorized by resection extent as total gastrectomy (TG), distal gastrectomy (DG), or bypass-only operation.

### 2.3. Outcome Definitions

Postoperative outcomes were systematically assessed using the Clavien–Dindo (C-D) grading system, a validated and internationally recognized classification for standardized surgical complication reporting [15]. Any adverse event occurring within 30 days of surgery or during the index hospitalization—whichever was longer—was captured and graded accordingly. Captured events encompassed anastomotic leakage or stricture, duodenal stump leakage, intra-abdominal or intraluminal hemorrhage, abdominal fluid collection or abscess, pancreatic fistula, mechanical ileus, wound-related complications, pneumonia, cerebrovascular events, and cardiac complications. Grades I and II reflect complications managed conservatively, without surgical, endoscopic, or radiological re-intervention. Severe morbidity was defined as a C-D grade of IIIa or higher, encompassing complications requiring interventional or operative management (grade IIIa–IIIb), single- or multi-organ dysfunction (grade IVa–IVb), and death (grade V). In-hospital mortality was defined as any death occurring within 30 days postoperatively or before discharge. To further delineate the influence of operative extent on perioperative risk, all outcomes were stratified by the type of resection performed: total gastrectomy (TG), distal gastrectomy (DG), and bypass-only procedures.

### 2.4. Statistical Analysis

Continuous variables are presented as the standardized means, with differences between means assessed via analysis of variance. Missing values were excluded from the calculations of means and standard deviations. Categorical variables are reported as counts and percentages, with frequency differences analyzed via the χ^2^ test. All the statistical analyses were conducted using IBM SPSS Statistics version 26.0 for Windows (IBM Corporation, Armonk, NY, USA) with statistical significance set at *p* < 0.05. Details of the statistical methods that were used are provided in the table footnotes.

## 3. Results

### 3.1. Patient Demographics

The demographic characteristics of the enrolled patients are shown in Table 1. In total, 12,420 patients were enrolled, and the mean age was 63 years. The numbers of males and females were 8193 and 4227, respectively, with a ratio of 1.9:1. The mean body mass index (BMI) of the patients was 23.9 ± 3.4 kg/m^2^, and the mean tumor size was 3.8 ± 2.8 cm. Among the TNM stages, stage I (*n* = 8410) was the most common, accounting for 67.7% of the cases, followed by stages II (*n* = 1899, 15.3%), III (*n* = 1805, 14.5%) and IV (*n* = 306, 2.5%). Preoperative chemotherapy was performed in 3.5% of the enrolled patients, and conversion surgery was performed in 0.5% (*n* = 61) of the patients. Postoperative chemotherapy was performed in 25% of the patients, and comorbidities coexisted in 64.1% of the patients before gastrectomy. For the American Society of Anesthesiologists (ASA) score distribution, an ASA score of 2 was the most common and accounted for 61.2% of the patients, an ASA score of 1 was observed in 22.5% of the patients, an ASA score of 3 was given to 15.8% of the patients, and ASA scores of 4 and 5 accounted for 0.4% and 0.1% of the patients, respectively. The mean hospital stay was 9.7 ± 10.2 days. With respect to approach methods, intracorporeal anastomosis (totally laparoscopic gastrectomy) was performed in 57.6% of the patients, extracorporeal anastomosis (laparoscopy-assisted gastrectomy) in was performed in 8.9% of the patients, robotic gastrectomy was performed in 5.5% of the patients, and open gastrectomy was performed in 27.8% of the patients. The extent of gastric resection varied among patients; distal gastrectomies were performed in 78% of the patients, total gastrectomies were performed in 21.2% of the patients, and bypass surgeries were performed in only 0.8% of the patients. In terms of curability, R0 was achieved in 97.5% of the cases, whereas R1, R2, and R3 accounted for 0.4%, 1.3%, and 0.8% of the cases, respectively. Complications occurred in 13.53% of the enrolled patients; severe complications of C-D grade IIIa or higher occurred in 4.53% of the patients, and death occurred in 0.2% of the patients.

### 3.2. Comparison Between the Curative Gastrectomy (CG) Group and the Palliative Surgery (PS) Group

Data comparing the features between the curative gastrectomy and palliative surgery groups are shown in Table 2. In the comparison between the two groups, the age, sex, comorbidities, and number of harvested lymph nodes were not significantly different. The BMI values were significantly greater in the CG group (24.0 kg/m^2^ ± 3.3) than in the PS group (22.0 kg/m^2^ ± 3.5) (*p* < 0.001). Tumor sizes were significantly greater in the PS group (8.4 cm ± 4.9) than in the CG group (3.7 cm ± 2.7) (*p* < 0.001).

With respect to the TNM stage, stages I–III were predominant in the CG group, whereas Stage IV was only observed in the PS group (*p* < 0.001). Preoperative chemotherapy was performed in 8.2% of the PS group patients, and conversion surgery was performed in 8.5% of PS group patients, which was significantly greater than the proportion of procedures performed on patients in the CG group. The ASA scores were significantly different, with the CG group having higher proportions of patients with ASA 2 designations, while the PS group had higher proportions of patients with ASA scores of 3 and 4.

The durations of hospital stays were significantly longer in the PS group (14.0 days ± 13.0) than in the CG group (9.6 days ± 10.1) (*p* < 0.001). Regarding the approach methods, the CG group included 27% open methods and 73% minimally invasive surgeries (58.4% totally laparoscopic gastrectomies, 9.1% laparoscopy-assisted gastrectomies, and 2.8% robotic surgeries). In contrast, 62.4% of the patients in the PS group underwent open methods and 37.6% underwent minimally invasive approaches (28.1% totally laparoscopic gastrectomies, 1.3% laparoscopy-assisted gastrectomies, 0.3% robotic surgeries, and 7.8% laparoscopic biopsies) (*p* < 0.001).

The C-D classifications were statistically different between the CG and PS groups, with scores of 0–1 being more prevalent in the CG group and scores of II–V more prevalent in the PS group (*p* < 0.001). The severe morbidity rate (C-D ≥ IIIa) was significantly greater in the PS group (10.2%) than in the CG group (4.8%) (*p* < 0.001). The mortality rate was also significantly greater in the PS group (1.6%) than in the CG group (0.2%) (*p* = 0.001).

### 3.3. Comparison of the Types of Severe Postoperative Complications (CD > IIIa) Between the CG and PS Groups

The types of severe complications (C-D ≥ IIIa) were compared between the curability groups (Table 3). The incidence of leakage was significantly greater in the PS group (3.9%) than in the CG group (1.3%) (*p* = 0.001). The incidence of pancreatic fistula was also significantly greater in the PS group (1%) than in the CG group (0.2%) (*p* = 0.036). Other complications did not significantly differ.

### 3.4. Comparison of Severe Postoperative Complications (CD > IIIa) and Mortality According to the Extent of Resection

The incidence of severe complications differed according to the extent of gastric resection (Table 3). In patients who underwent distal gastrectomies (DGs), the incidence of severe complications was significantly greater in the PS group (13%) than in the CG group (3.8%) (*p* < 0.001). For total gastrectomies (TGs), the incidence of severe complications was greater in the PS group (14.3%) than in the CG group (8.5%), but the difference was not statistically significant (*p* = 0.085). There was no significant difference in mortality rates between the CG (0.2%) and PS groups (0.9%) for DGs (*p* = 0.171). However, for TGs, there was a significant difference between the CG (0.3%) and PS groups (3.3%) (*p* = 0.006).

### 3.5. Comparison of Severe Postoperative Complications (CD > IIIa) and Mortality According to the Extent of Resection in the Palliative Group

Within the PS group, the incidence of severe complications differed significantly according to the extent of resection (Table 4 and Table 5). Compared with bypass only (3.8%), both DGs (13%) and TGs (14.3%) had significantly higher severe complication rates (*p* = 0.027). Patient mortality was greater for TGs (3.3%) than bypass only (1%) and DG procedures (0.9%), although the difference was not statistically significant (*p* = 0.334).

### 3.6. Logistic Regression Analysis of Severe Complications and Mortality

To identify independent predictors of perioperative outcomes across the entire cohort, univariable and multivariable logistic regression analyses were performed for two primary endpoints: severe postoperative complications (C-D ≥ IIIa) and perioperative mortality (C-D grade V). Results are presented in Table 6 and Table 7, respectively.

In multivariable analysis, independent predictors of severe complications included advancing age (OR 1.01, 95% CI 1.00–1.02, *p* < 0.001), higher TNM stage (Stage II: OR 1.51, 95% CI 1.18–1.94; Stage III: OR 2.13, 95% CI 1.68–2.70; both *p* < 0.001), ASA score of 5 (OR 4.16, 95% CI 1.03–16.7), open gastrectomy (OR 1.36, 95% CI 1.10–1.67, *p* = 0.028), and R2 residual disease (OR 1.80, 95% CI 0.76–4.24, *p* = 0.036). Combined organ resection was independently associated with a paradoxically lower risk of severe complications (OR 0.51, 95% CI 0.41–0.65, *p* < 0.001), likely reflecting a positive selection effect. For perioperative mortality, advancing age (OR 1.08, 95% CI 1.03–1.12), neoadjuvant chemotherapy (OR 2.39, 95% CI 1.09–5.23, *p* = 0.029), and ASA score of 5 (OR 36.4, 95% CI 2.52–527.8) were the strongest independent predictors, while TNM stage did not retain independent prognostic significance after multivariable adjustment.

## 4. Discussion

The primary aim of this study was to compare the incidences of severe complications and mortality rates between palliative and curative gastric cancer surgeries utilizing data from a large-scale nationwide survey. Our findings revealed that patients who underwent palliative surgery experienced significantly higher rates of severe complications (10.2% vs. 4.8%, *p* < 0.001) and mortality (1.6% vs. 0.2%, *p* = 0.001) than those who underwent curative surgery. To the best of our knowledge, this is the first study to analyze the differences in severe complication rates and mortality between palliative and curative gastric cancer surgeries using data from a large-scale nationwide survey.

Compared with patients in the CG group, the significantly lower BMI, larger tumor size, and higher ASA scores (3 and 4) of patients in the PS group indicate the advanced disease burden of these patients (Table 2). The poorer preoperative condition of patients in the PS group, as indicated by these parameters, likely contributed to the higher incidence of severe complications observed in this cohort. The significantly prolonged hospital stays observed in the PS group can be attributed to the higher frequency of severe complications in this cohort. This observation is supported by several previous studies. With respect to BMI, a 2018 study of gastric cancer patients revealed that a low BMI was associated with increased severe postoperative complications, particularly in patients with stage III/IV disease [16]. The study also revealed that low BMI was an independent risk factor for severe postoperative complications in the stage III/IV subgroup. Another study published in 2015 demonstrated that a low BMI was associated with more severe postoperative complications and poorer prognosis in gastric cancer patients undergoing gastrectomy [17]. Tumor size has also been identified as a significant factor in postoperative complications. A 2012 study demonstrated that tumor size is an independent prognostic factor in advanced gastric cancer, irrespective of serosal invasion [18]. The study revealed that a larger tumor size was associated with a poorer prognosis and increased risk of complications. Furthermore, a 2024 study showed that a tumor size > 5 cm was significantly associated with an elevated rate of complications via univariate analysis [19]. The impact of the ASA score on surgical outcomes has been well documented in gastric cancer patients. A 2022 study revealed that ASA classes III-IV were significantly associated with increased postoperative complications via univariate analysis, although this association did not persist when evaluated using multivariate analysis [20]. Additionally, a 2023 study reported that the ASA score, along with other factors such as age and Eastern Cooperative Oncology Group (ECOG) score, was a statistically significant risk factor for severe complications, as demonstrated via multivariate analysis [7].

The significantly higher incidence of pancreatic fistula in the PS group (1.0% vs. 0.2%, *p* = 0.036) is mechanistically explained by the substantially higher rates of combined organ resection (18.6% vs. 9.3%, *p* < 0.001) and pancreas-specific combined surgery (4.2% vs. 0.6%, *p* < 0.001) observed in this group (Table 2). The larger tumor burden in PS patients (mean 8.4 cm vs. 3.7 cm) and advanced local invasion characteristic of Stage IV disease frequently necessitate direct pancreatic involvement—whether through formal distal pancreatectomy or extensive peripancreatic dissection—substantially elevating the risk of pancreatic ductal injury and subsequent fistula formation. Furthermore, the poorer nutritional status of PS patients, reflected in their significantly lower BMI (22.0 vs. 24.0 kg/m^2^, *p* < 0.001), impairs tissue healing and pancreatic fistula containment. The higher proportion of open surgery in the PS group (62.4% vs. 27.0%) may further contribute through greater tissue manipulation in the peripancreatic region. These findings collectively underscore the importance of preoperative assessment of pancreatic involvement and nutritional optimization when palliative gastrectomy with combined resection is contemplated.

The REGATTA trial demonstrated no significant difference in overall survival between patients who underwent palliative gastrectomy followed by chemotherapy and those who received chemotherapy alone for advanced gastric cancer with a single noncurable factor (median survival 14.3 months vs. 16.6 months, HR 1.09, 95% CI 0.78–1.52) [9]. However, it is important to note that the REGATTA trial did not directly compare complication rates between palliative and curative gastrectomy operations. A key finding from the REGATTA trial was the poor survival outcomes in patients with upper-third gastric tumors who underwent TG, likely due to decreased chemotherapy compliance postsurgery. This observation aligns with our study results, where we observed significantly higher mortality rates for patients who received TGs in the PS group (3.3%) than in the CG group (0.3%) (*p* = 0.006). These findings collectively suggest that palliative TG for stage IV gastric cancer should be approached with great caution.

Despite the substantially elevated perioperative risk demonstrated in this study, palliative gastrectomy may confer meaningful clinical benefit in carefully selected subgroups of patients with Stage IV gastric cancer. Patients with limited peritoneal dissemination and without systemic organ metastases may derive particular benefit, as gastrectomy in this setting has been associated with improved overall survival compared with non-surgical management, likely through enhanced chemotherapy compliance following primary tumor reduction [21,22]. For patients with gastric outlet obstruction refractory to endoscopic stenting, surgical gastrojejunostomy offers more durable palliation with a lower rate of reintervention compared to repeated endoscopic procedures, albeit at the cost of greater short-term morbidity [23]. Furthermore, patients who demonstrate a robust oncological response to induction chemotherapy may be candidates for conversion surgery—a strategy that has been associated with significantly prolonged survival, particularly when R0 resection is achievable [24,25]. Ultimately, the decision to pursue palliative gastrectomy should be made through a structured multidisciplinary team process incorporating the patient’s performance status, nutritional reserve, biomarker profile, and anticipated tolerability of postoperative systemic therapy [21,26]. Our findings—severe complication and mortality rates of 10.2% and 1.6%, respectively—underscore that these risks may be justifiable only in appropriately selected patients when weighed against the potential for meaningful symptom relief and improved oncological outcomes.

A recent meta-analysis reported improved overall survival in patients with advanced gastric cancer who underwent palliative gastrectomy (HR: 1.49; 95% CI: 1.12–1.99; *p* = 0.006) [12]. This contrasts with the REGATTA trial results and highlights the ongoing debate in this field. This discrepancy may be due to differences in patient selection, surgical techniques, or postoperative management across studies. Given these conflicting findings, the decision to perform palliative gastrectomy in stage IV gastric cancer patients should be made carefully, and the potential risks and benefits for each individual patient should be evaluated [21,27]. Factors such as tumor location, patient performance status, and the ability to tolerate postoperative chemotherapy should be thoroughly considered [10]. Further research is needed to identify subgroups of patients who may benefit from palliative gastrectomy and to optimize perioperative management strategies to improve outcomes.

The comparable mean number of harvested lymph nodes between the PS and CG groups (39.4 vs. 38.9, *p* = 0.71) warrants further discussion. In the Korean surgical context, D2 lymphadenectomy represents the established standard of care at high-volume centers and is routinely performed regardless of surgical intent, particularly when formal gastrectomy is undertaken [4,28]. Furthermore, a substantial proportion of cases in the PS group may have been initiated as curative resections but were reclassified as palliative intraoperatively—upon discovery of peritoneal seeding, positive cytology, or distant metastasis—at which point D2 lymphadenectomy had already been completed. This phenomenon of intraoperative upstaging is well recognized in gastric cancer surgery and likely accounts for the unexpectedly extensive lymphadenectomy observed in this palliative cohort [3,29]. As the KGCA dataset captures actual intraoperative surgical findings rather than preoperative intent, the high lymph node yield reflects the surgical reality of procedures completed as palliative rather than those planned as such from the outset.

This study has several limitations that warrant careful consideration. First, the retrospective design of the KGCA nationwide survey precluded additional data acquisition or prospective validation; causal inferences should therefore be interpreted with appropriate caution. Second, the KGCA 2019 dataset does not systematically capture individualized preoperative clinical indications for palliative surgery on a case-by-case basis. Consequently, it was not possible to distinguish among the heterogeneous clinical scenarios that prompted palliative intervention—such as gastric outlet obstruction refractory to endoscopic stenting, uncontrolled tumor hemorrhage, perforation, or conversion surgery following chemotherapy response—nor to assess whether less invasive alternatives were considered or attempted prior to surgery. Surgical intent was therefore classified based on pathological staging and resection margin status (R0 for curative; R1–R3 for palliative), which, while objective and reproducible, does not fully reflect the complexity of preoperative clinical decision-making. This precludes a more granular analysis of how differing surgical indications may have influenced postoperative outcomes. Future nationwide registry designs should incorporate structured data fields for surgical indications to enable more clinically precise comparative analyses in this high-risk population. Third, while mortality was defined in accordance with standard surgical outcome criteria—as death occurring within 30 days postoperatively or during the same hospitalization—the KGCA dataset does not systematically differentiate between deaths directly attributable to surgical complications (e.g., anastomotic leakage leading to refractory sepsis) and those resulting from rapid disease progression in the immediate postoperative period. This distinction is particularly challenging in the palliative setting, where the clinical boundary between surgical morbidity and underlying oncological deterioration is often difficult to delineate; the reported mortality figures should therefore be interpreted as reflecting overall perioperative mortality rather than surgery-specific mortality. Fourth, the substantially smaller PS group (*n* = 306) compared to the CG group (*n* = 12,114) limits the statistical power of subgroup analyses, and the significant baseline imbalances between groups—including disease stage, tumor size, BMI, ASA score, and surgical approach—represent potential confounders that could not be fully adjusted for in the present analysis. Fifth, as a nationwide multicenter study encompassing 68 institutions, variability in surgical techniques, anastomotic methods, and perioperative management protocols may have introduced institutional heterogeneity that was not possible to control for. Sixth, the KGCA dataset does not systematically record whether anastomoses were hand-sewn or stapled, or whether reinforcing sutures were placed, precluding a detailed technical analysis of anastomotic outcomes. Furthermore, the KGCA 2019 nationwide dataset does not systematically capture data on targeted therapy or immunotherapy regimens administered in conjunction with surgery. Specifically, information on HER-2 status, trastuzumab use in HER-2-positive patients, or PD-1/PD-L1 immune checkpoint inhibitor administration was not recorded as structured fields in the standardized case report form, as the survey was primarily designed to capture surgical techniques and short-term postoperative outcomes. It should be noted, however, that in 2019, immune checkpoint inhibitors had not yet been approved as a standard first-line treatment option for gastric cancer in Korea; the prevailing systemic regimen consisted of XELOX- or FOLFOX-based chemotherapy, with trastuzumab reserved for HER-2-positive cases. As such, the absence of immunotherapy data is unlikely to have materially influenced the perioperative outcomes analyzed in this study, but may limit the generalizability of our findings to contemporary practice, in which immune checkpoint inhibitors have since become integral to the systemic management of advanced gastric cancer [30]. Despite these limitations, to the best of our knowledge, this study represents the largest comparative analysis of severe complication and mortality rates between palliative and curative gastric cancer surgeries to date, providing nationally representative evidence that may meaningfully inform surgical decision-making in this high-risk population.

Finally, the incidence of severe complications and mortality was significantly higher in the PS group, particularly for anastomotic leakage and pancreatic fistula. Analysis by resection extent revealed higher rates of complications and mortality for total gastrectomies than for distal gastrectomies (severe morbidity rate: DG 346/9183 [3.8%] vs. TG 14/108 [13%], *p* < 0.001; mortality: DG 8/2445 [0.3%] vs. TG 3/91 [3.3%], *p* = 0.006). These findings underscore the importance of thoroughly informing patients about the risks of palliative surgery and exercising particular care to prevent and manage complications when palliative gastrectomy is necessary. A key constraint of the present study is the absence of structured data on preoperative surgical indications within the KGCA dataset. As individualized clinical triggers for palliative intervention—including gastric outlet obstruction, tumor bleeding, and perforation—were not captured in the standardized case report form, the relative contribution of each indication to the observed complication and mortality rates could not be determined. This underscores an important direction for future registry development: the prospective incorporation of structured surgical indication fields would allow investigators to determine not only whether palliative surgery was performed, but why, thereby enabling more clinically meaningful risk stratification and outcome comparisons in patients with Stage IV gastric cancer.

## 5. Conclusions

In this nationwide study, palliative surgery for gastric cancer was associated with significantly higher rates of severe postoperative complications and mortality than curative gastrectomy. In particular, anastomotic leakage and pancreatic fistula occurred more frequently in the palliative surgery group, and total gastrectomy was associated with a particularly high mortality risk in the palliative setting. These findings suggest that palliative gastric cancer surgery is accompanied by a substantial perioperative risk.

Accordingly, the indication for palliative surgery in patients with gastric cancer should be determined with caution. Careful patient selection, comprehensive preoperative assessment, and thorough discussion of the expected risks and potential benefits are essential when considering surgical palliation. Further efforts to optimize perioperative management may help reduce major morbidity and mortality in this high-risk population.

## Figures and Tables

**Table 1:** Patients demographics.

Factor		Value
Age (years)		63.0 ± 11.8
Sex	Male	8193 (66%)
	Female	4227 (34%)
BMI (kg/m^2^)		23.9 ± 3.4
Histologic subtype	Papillary carcinoma	81 (0.7%)
	Well differentiated tubular adenocarcinoma	1261 (10.2%)
	Moderate differentiated tubular adenocarcinoma	4064 (32.9%)
	Poorly differentiated tubular adenocarcinoma	3322 (26.9%)
	Poorly cohesive carcinoma	2324 (18.8%)
	Mucinous carcinoma	174 (1.4%)
	Mixed type with Signet ring cell carcinoma	778 (6.3%)
	Gastric carcinoma with lymphoid stroma	161 (1.3%)
	Others	172 (1.4%)
Tumor size (cm)		3.8 ± 2.8
TNM stage	I	8410 (67.7%)
	II	1899 (15.3%)
	III	1805 (14.5%)
	IV	306 (2.5%)
Pre-operative Chemotherapy	No	11,795 (95%)
	Yes	440 (3.5%)
	Conversion surgery	61 (0.5%)
Post-operative Chemotherapy	No	8594 (75%)
	Yes	2865 (25%)
Existence of Comorbidity	No	4064 (35.9%)
	Yes	7250 (64.1%)
ASA score	1	2734 (22.5%)
	2	7447 (61.2%)
	3	1916 (15.8%)
	4	53 (0.4%)
	5	12 (0.1%)
Hospital stay (days)		9.7 ± 10.2
Approach methods	Totally laparoscopic gastrectomy	7098 (57.6%)
	Laparoscopy-assisted gastrectomy	1092 (8.9%)
	Open gastrectomy	3429 (27.8%)
	Robot-assisted gastrectomy	677 (5.5%)
Gastric resection extent	Distal gastrectomy	9686 (78%)
	Total gastrectomy	2629 (21.2%)
	Bypass only	105 (0.8%)
Number of harvested lymph nodes		38.9 ± 17.9
Curability	R0	12,114 (97.5%)
	R1	45 (0.4%)
	R2	157 (1.3%)
	R3	104 (0.8%)
Clavien–Dindo classification	I	256 (2.1%)
	II	859 (6.9%)
	IIIa	318 (2.6%)
	IIIb	146 (1.2%)
	IVa	90 (0.7%)
	IVb	4 (0.03%)
	V (Mortality)	28 (0.2%)

ASA, American Society for Anesthesiology; Values are represented as mean ± standard deviation or number of patients (percentages). Values and percentages were analyzed after excluding missing values. R0 indicates no residual tumor (curative resection); R1 indicates microscopic residual tumor at the resection margin; R2 indicates macroscopic residual tumor at the primary site (grossly incomplete resection); and R3 indicates distant metastasis, peritoneal seeding, or positive peritoneal cytology at the time of surgery, regardless of the extent of primary resection.

**Table 2:** Comparison of patient variable between curative gastrectomy (CG) group and Palliative surgery (PS) group.

Factors		CG (*n* = 12,114)	PS (*n* = 306)	*p* Value
Age (years)	(*n* = 11,638 vs. 298)	63.0 ± 11.7	64.1 ± 12.8	*p* = 0.09
Sex	Male	7983 (65.9%)	210 (68.6%)	*p* = 0.32
	Female	4131 (34.1%)	96 (31.4%)	
BMI (kg/m^2^)	(*n* = 12,103 vs. 304)	24.0 ± 3.3	22.0 ± 3.5	*p* < 0.001
Tumor size (cm)	(*n* = 11,915 vs. 229)	3.7 ± 2.7	8.4 ± 4.9	*p* < 0.001
Histologic subtype	Papillary carcinoma	8 (0.7%)	1 (0.4%)	*p* < 0.001
	Well differentiated tubular adenocarcinoma	1255 (10.4%)	6 (2.4%)	
	Moderate differentiated tubular adenocarcinoma	3997 (33.1%)	67 (26.4%)	
	Poorly differentiated tubular adenocarcinoma	3235 (26.8%)	87 (34.3%)	
	Poorly cohesive carcinoma	2262 (18.7%)	62 (24.4%)	
	Mucinous carcinoma	167 (1.4%)	7 (2.8%)	
	Mixed type with Signet ring cell carcinoma	776 (6.4%)	9 (3.5%)	
	Gastric carcinoma with lymphoid stroma	157 (1.3%)	4 (1.6%)	
	Others	161 (1.3%)	11 (4.3%)	
TNM stage	I	8410 (69.4%)		*p* < 0.001
	II	1899 (15.7%)		
	III	1805 (14.9%)		
	IV	0	306 (100%)	
Pre-operative Chemotherapy	No	11,540 (96.2%)	255 (83.5%)	*p* < 0.001
	Yes	415 (3.5%)	25 (8.2%)	
	Conversion surgery	35 (0.3%)	26 (8.5%)	
Post-operative Chemotherapy	No	8489 (76%)	105 (35.5%)	*p* < 0.001
	Yes	2674 (24%)	191 (64.5%)	
Existence of Comorbidity		7061 (64.1%)	189 (64.5%)	*p* = 0.90
ASA score	1	2667 (22.5%)	67 (22.3%)	*p* < 0.001
	2	7286 (61.4%)	161 (53.7%)	
	3	1853 (15.6%)	63 (21%)	
	4	46 (0.4%)	7 (2.3%)	
	5	10 (0.1%)	2 (0.7%)	
Hospital stay (days)	(*n* = 12,106 vs. 303)	9.6 ± 10.1	14.0 ± 13.0	*p* < 0.001
Approach methods	Totally laparoscopic gastrectomy	7012 (58.4%)	86 (28.1%)	*p* < 0.001
	Laparoscopy-assisted gastrectomy	1088 (9.1%)	4 (1.3%)	
	Open gastrectomy	3238 (27%)	191 (62.4%)	
	Robotic gastrectomy	676 (2.8%)	1 (0.3%)	
	Laparoscopic Biopsy only		24 (7.8%)	
Gastric resection extent	Distal gastrectomy	9577 (77.8%)	109 (35.6%)	*p* < 0.001
	Total gastrectomy	2537 (20.9%)	92 (30.1%)	
	No resection (Bypass only)	0	105 (34.3%)	
Number of harvested lymph nodes	(*n* = 12,104 vs. 198)	38.9 ± 17.8	39.4 ± 20.5	*p* = 0.71
Combined resection		1106/12,114 (9.3%)	57/306 (18.6%)	*p* < 0.001
Pancreas combined surgery		76/12,114 (0.6%)	13/306 (4.2%)	*p* < 0.001
Curability	R0	12,114 (100%)	0	
	R1		45 (14.7%)	
	R2		157 (51.3%)	
	R3		104 (34%)	
Clavien–Dindo classification	0	9992 (85.9%)	239 (78.6%)	*p* < 0.001
	I	253 (2.2%)	3 (1.0%)	
	II	828 (7.1%)	31 (10.2%)	
	IIIa	303 (2.6%)	15 (4.9%)	
	IIIb	140 (1.2%)	6 (2.0%)	
	IVa	86 (0.7%)	4 (1.3%)	
	IVb	3 (0.02%)	1 (0.3%)	
	V	23 (0.2%)	5 (1.6%)	
Severe Morbidity rate (Clavien–Dindo ≥ IIIa)		555/11,628 (4.8%)	31/304 (10.2%)	*p* < 0.001
Mortality (Clavien–Dindo V)		23/11,628 (0.2%)	5/304 (1.6%)	*p* = 0.001

Severe morbidity means Clavien–Dindo classification IIIa or higher; ASA, American Society for Anesthesiology. The difference in categorical variables in their frequency was implemented using Chi-square analysis.

**Table 3:** Comparison of the types of postoperative severe complication (Clavien–Dindo ≥ IIIa) between curative gastrectomy (CG) group and Palliative surgery (PS) group.

Complication (Total *n* = 12,244)	CG	PS	*p* Value
Anastomosis leakage	157 (1.3%)	12 (3.9%)	0.001
Anastomosis stricture	70 (0.6%)	1 (0.3%)	1.0
bleeding	102 (0.9%)	6 (2%)	0.057
Pancreatic fistula	26 (0.2%)	3 (1%)	0.036
Intraabdominal abscess	269 (2.3%)	7 (2.3%)	1.0
Wound problem	157 (1.3%)	7 (2.3%)	0.201
Mechanical ileus	145 (1.2%)	5 (1.6%)	0.435
Pneumonia	208 (1.8%)	10 (0.3%)	0.075
CVA	11 (0.1%)	0	1.0
Heart problem	44 (0.4%)	0	0.630
Others	494 (4.2%)	14 (4.6%)	0.666

CVA, cerebrovascular accident; Difference in categorical variables in their frequency was implemented using Chi-square analysis.

**Table 4:** Comparison of postoperative severe complication (Clavien–Dindo > IIIa) and mortality according to resection extent.

Complication	Resection Extent	CG	PS	*p* Value
Severe Morbidity rate (Clavien–Dindo ≥ IIIa)	DG	346/9183 (3.8%)	14/108 (13%)	<0.001
	TG	209/2445 (8.5%)	13/91 (14.3%)	0.085
Mortality (Clavien–Dindo V)	DG	15/9183 (0.2%)	1/108 (0.9%)	0.171
	TG	8/2445 (0.3%)	3/91 (3.3%)	0.006

CG, Curative gastrectomy; PS, Palliative surgery; Difference in categorical variables in their frequency was implemented using Chi-square analysis.

**Table 5:** Comparison of postoperative severe complication (Clavien–Dindo > IIIa) and mortality according to resection extent in palliative surgery (PS) group.

Complication	DG	TG	Bypass Only	*p* Value
Severe Morbidity rate (Clavien–Dindo ≥ IIIa)	14/108 (13%)	13/91 (14.3%)	4/105 (3.8%)	0.027
Mortality (Clavien–Dindo V)	1/108 (0.9%)	3/91 (3.3%)	1/105 (1%)	0.334

DG, distal gastrectomy; TG, total gastrectomy; Difference in categorical variables in their frequency was implemented using Chi-square analysis.

**Table 6:** Univariate and multivariate analysis of postoperative severe complication (Clavien–Dindo ≥ IIIa).

Risk Factor		Univariate			Multivariate		
		OR	95% CI	*p* Value	OR	95% CI	*p* Value
Age		1.02	1.01–1.03	<0.001	1.01	1.00–1.02	<0.001
BMI		0.99	0.97–1.01	0.693			
Histologic subtype	Papillary carcinoma	1		<0.001	1		0.001
	Well differentiated tubular adenocarcinoma	0.32	0.15–0.66		0.54	0.25–1.16	
	Moderate differentiated tubular adenocarcinoma	0.41	0.21–0.81		0.51	0.24–1.07	
	Poorly differentiated tubular adenocarcinoma	0.31	0.15–0.62		0.36	0.17–0.75	
	Poorly cohesive carcinoma	0.23	0.11–0.46		0.32	0.15–0.69	
	Mucinous carcinoma	0.32	0.12–0.85		0.24	0.08–0.75	
	Mixed type with Signet ring cell carcinoma	0.33	0.15–0.70		0.48	0.21–1.06	
	Gastric carcinoma with lymphoid stroma	0.29	0.10–0.81		0.30	0.09–0.95	
	Others	0.30	0.11–0.84		0.24	0.07–0.79	
TNM Stage	I	1		<0.001	1		<0.001
	II	1.81	1.46–2.24		1.512	1.18–1.94	
	III	2.05	2.05–3.03		2.13	1.68–2.70	
	IV	1.11	1.11–2.22		2.21	0.81–6.06	
Neoadjuvante CTx		1.42	1.10–1.83	0.006			0.057
ASA score	1	1		<0.001	1		0.032
	2	1.10	0.89–1.35		0.91	0.72–1.15	
	3	1.87	1.47–2.39		1.22	0.92–1.62	
	4	1.64	0.58–4.60		0.94	0.32–2.77	
	5	11.5	3.41–38.70		4.16	1.03–16.7	
Approach method	Totally laparoscopic gastrectomy	1		<0.001	1		0.028
	Laparoscopy-assisted gastrectomy	1.01	0.733–1.39		0.92	0.63–1.33	
	Open gastrectomy	1.89	1.60–2.24		1.36	1.10–1.67	
	Robot assisted gastrectomy	0.63	0.40–0.99		0.84	0.51–1.38	
	No resection (Bypass only)	0.56	0.24–1.27				
Combined resection		0.42	0.34–0.52	<0.001	0.51	0.41–0.65	<0.001
Curability	R0	1		<0.001	1		0.036
	R1	1.07	0.47–2.45		0.18	0.02–1.71	
	R2	3.76	2.45–5.77		1.80	0.76–4.24	
	R3	0.43	0.43–1.20		0.50	0.08–2.91	

**Table 7:** Univariate and multivariate analysis of postoperative mortality (Clavien–Dindo grade V).

Risk Factor		Univariate			Multivariate		
		OR	95% CI	*p* Value	OR	95% CI	*p* Value
Age		1.11	1.07–1.15	<0.001	1.08	1.03–1.12	<0.001
BMI		0.87	0.78–0.96	0.010			0.352
Histologic subtype				0.653			
TNM Stage	I	1		0.018			0.790
	II	2.42	0.96–6.04				
	III	3.11	1.32–7.29				
	IV	3.96	1.28–12.18				
Neoadjuvante CTx		2.56	1.27–5.14	0.008	2.39	1.09–5.23	0.029
ASA score	1	1		<0.001	1		0.007
	2	2.29	0.51–10.26		1.41	0.31–6.46	
	3	14.17	3.29–60.92		4.11	0.90–18.7	
	4	25.40	2.27–284.08		4.66	0.37–58.7	
	5	136.27	11.49–1614.92		36.4	2.52–527.8	
Approach method	Totally laparoscopic gastrectomy	1		0.033			0.932
	Laparoscopy-assisted gastrectomy	0.48	0.064–3.70				
	Open gastrectomy	2.83	1.42–5.67				
	Robot assisted gastrectomy	-					
	No resection (Bypass only)	2.12	0.27–16.21				
Combined resection		0.28	0.13–0.61	0.001	0.36	0.16–0.81	0.014
Curability	R0	1		<0.001			0.223
	R1	4.04	0.54–30.0				
	R2	11.74	4.06–33.9				
	R3	3.12	0.94–10.3				

## Data Availability

The datasets generated and analyzed during the current study are not publicly available due to restrictions from the Korean Gastric Cancer Association, which owns the dataset. However, the data are available from the corresponding author, upon reasonable request and with permission of the Korean Gastric Cancer Association.
